# Golgi Disruption and Early Embryonic Lethality in Mice Lacking USO1

**DOI:** 10.1371/journal.pone.0050530

**Published:** 2012-11-21

**Authors:** Susie Kim, Adele Hill, Matthew L. Warman, Patrick Smits

**Affiliations:** 1 Orthopaedic Research Laboratories, Department of Orthopaedic Surgery, Boston Children's Hospital, Boston, Massachusetts, United States of America; 2 Howard Hughes Medical Institute, Boston Children's Hospital, Boston, Massachusetts, United States of America; NHLBI, NIH, United States of America

## Abstract

Golgins are a family of long rod-like proteins characterized by the presence of central coiled-coil domains. Members of the golgin family have important roles in membrane trafficking, where they function as tethering factors that capture transport vesicles and facilitate membrane fusion. Golgin family members also have essential roles in maintaining the organization of the Golgi apparatus. Knockdown of individual golgins in cultured cells resulted in the disruption of the Golgi structure and the dispersal of Golgi marker proteins throughout the cytoplasm. However, these cellular phenotypes have not always been recapitulated *in vivo*. For example, embryonic development proceeds much further than expected and Golgi disruption was observed in only a subset of cell types in mice lacking the ubiquitously expressed golgin GMAP-210. Cell-type specific functional compensation among golgins may explain the absence of global cell lethality when a ubiquitously expressed golgin is missing. In this study we show that functional compensation does not occur for the golgin USO1. Mice lacking this ubiquitously expressed protein exhibit disruption of Golgi structure and early embryonic lethality, indicating that USO1 is indispensable for early embryonic development.

## Introduction

Communication between different membrane bound compartments within a eukaryotic cell and between the cell and its extracellular environment is accomplished in large part by the membrane trafficking process (also known as vesicular trafficking). Two major but interconnecting trafficking pathways exist within the cell. The exocytic pathway is responsible for transporting proteins and lipids from their place of synthesis to their site of function in or outside the cell. The endocytic, or retrograde, pathway is responsible for transport of internalized cargo from the extracellular environment towards endosomes and lysosomes. The endocytic pathway is also responsible for the recycling of proteins involved in exocytic transport [1], [2], [3], [4].

Membrane trafficking occurs in four steps. (**1**) Cargo is selected, and a vesicular or tubular transport intermediate forms at a donor compartment. (**2**) The transport intermediate is delivered to the target membrane using molecular motors that travel along the cell's microtubule or actin filament system. (**3**) Tethering brings the transport intermediate and target membranes in close proximity. (**4**) Fusion of the two membranes leads to the transfer of cargo [5].

Because of its central role in cell function, membrane trafficking has been studied extensively. However, knowledge regarding the cell-type and tissue-specific aspects of membrane trafficking is incomplete. Few studies have addressed membrane trafficking in complex environments such as tissues or multi-cellular organisms. One insight derived from studying human membrane trafficking diseases is that mutations in components of the trafficking machinery thought to be of fundamental importance to the process and expected to have a global effect, instead result in cell-type-specific and/or cargo-specific phenotypes [6].

We previously reported that inactivation of the ubiquitously expressed membrane trafficking protein GMAP-210 (a.k.a. TRIP11) causes Achondrogenesis type 1A [7]. GMAP-210 is a member of the golgin protein family; these large coiled-coil proteins function as tethering factors that capture transport intermediates and aid in fusion with destination compartments. Golgins also provide structural support for the Golgi apparatus by tethering Golgi cisternae [8]. *In vitro* knockdown of GMAP-210 suggested it would be essential for maintenance of the Golgi stack structure [9]. However GMAP-210 deficiency *in vivo* did not cause early embryonic lethality, but instead affected the development of the skeleton [7]. Rats lacking another ubiquitously expressed golgin GIANTIN (a.k.a. Golgb1), thought to be essential for reformation of the Golgi stack structure after mitosis, also had a milder *in vivo* phenotype [10] than would have been predicted from *in vitro* studies [10], [11], [12].

The golgin USO1 (a.k.a. p115), which functionally interacts with GIANTIN, was also predicted to be essential for Golgi structure based on *in vitro* studies [11], [12], [13], [14], [15]. To investigate the *in vivo* consequence of USO1 deficiency we generated two independent mouse lines carrying gene traps (GT) in *Uso1*. Golgi disruption and death before embryonic day 8.5 occurred in homozygous mutant embryos from each gene-trap line indicating that USO1, unlike GMAP-210 and GIANTIN, is indispensable during early development.

**Figure 1:**
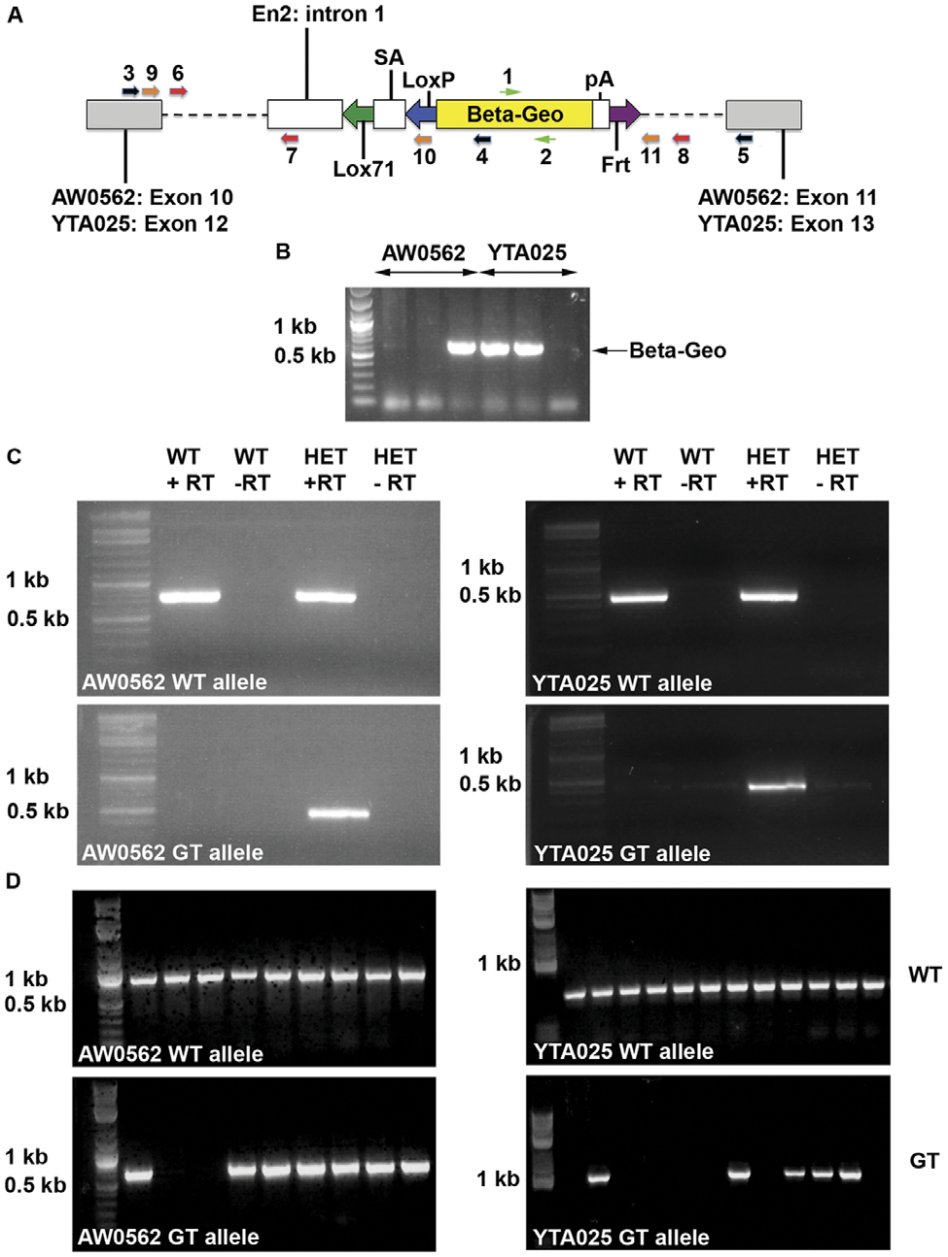
Generation of mice that transmit GT alleles at the *Uso1* locus. **A**) Schematic representation of the gene trap insertions into *Uso1*. The *Uso1* exons upstream and downstream of the GT are shaded grey. These are exons 10 and 11 for AW0562 and exons 12 and 13 for YTA025. The GT contains intron 1 and a strong splice acceptor (SA) from the engrailed locus (En2-intron1-SA), coding sequence for Beta-Geo, and a poly-A (pA) addition site. Sites that can be used for Cre-mediated (Lox71 and LoxP) and Flpe-mediated recombination (Frt) are also contained within the GT. Primer pairs used for RT-PCR and genotyping are numbered and color-coded and their approximate locations within the exon, intron, or GT vector are indicated. **B**) Genotyping of agouti offspring from chimeric males generated using AW0562 and YTA025 ES cells. Pups carrying the GT allele were identified by PCR amplification of a fragment of the Beta-Geo cassette (primer pair 1 – 2, green). **C**) RT-PCR confirming splicing of the GT to the *Uso1* gene in both cell lines (black primer pairs). RNA was extracted from primary skin fibroblasts established from wild-type (WT) and GT heterozygous (HET) mice. Top left panel: AW0562 wild-type (WT) allele, primer pair 3 – 5. Bottom left panel: AW0562 GT allele, primer pair 3 – 4. Top right panel: YTA025 WT allele, primer pair 3 – 5. Bottom right panel: YTA025 GT allele, primer pair 3 – 4. **D**) Genotyping of offspring from matings between wild type mice and AW0562 or YTA025 GT heterozygous mice confirming the insertion of the GT in the introns immediately following the trapped *Uso1* exons. Top left panel: AW0562 WT allele, primer pair 6 – 8 (red). Bottom left panel: AW0562 GT allele, primer pair 6 – 7 (red). Top right panel: YTA025 WT allele, primer pair 9 – 11 (orange). Bottom right panel: YTA025 GT allele, primer pair 9 – 10 (orange).

## Materials and Methods

### Generation of USO1 deficient mice

This study was carried out in strict accordance with the recommendations in the Guide for the Care and Use of Laboratory Animals of the National Institutes of Health. All animal experiments were completed under a protocol approved by the Institutional Animal Care and Use Committee of Children's Hospital Boston (Animal Welfare Assurance number: A3303-01).

Two *Uso1* GT ES cell lines, YTA025 (BayGenomics) and AW0562 (The Sanger Gene Trap Resources) were identified using the database of the International Gene Trap Consortium (www.genetrap.org) and obtained from the Mutant Mouse Regional Resource Center (www.mmrrc.org). ES cells were injected into 129SvE blastocysts by the Mouse Gene Manipulation Core of Boston Children's Hospital. Chimeric founder males were bred to wild-type C57BL/6 females (Jackson laboratories) and germline transmission was assessed by coat color. To confirm transmission of the *Uso1* GT allele, agouti offspring were genotyped by PCR for presence of the Beta-Geo selection cassette within the GT. GT heterozygous mice were maintained on a mixed 129SvE and C57BL/6 background.

**Figure 2:**
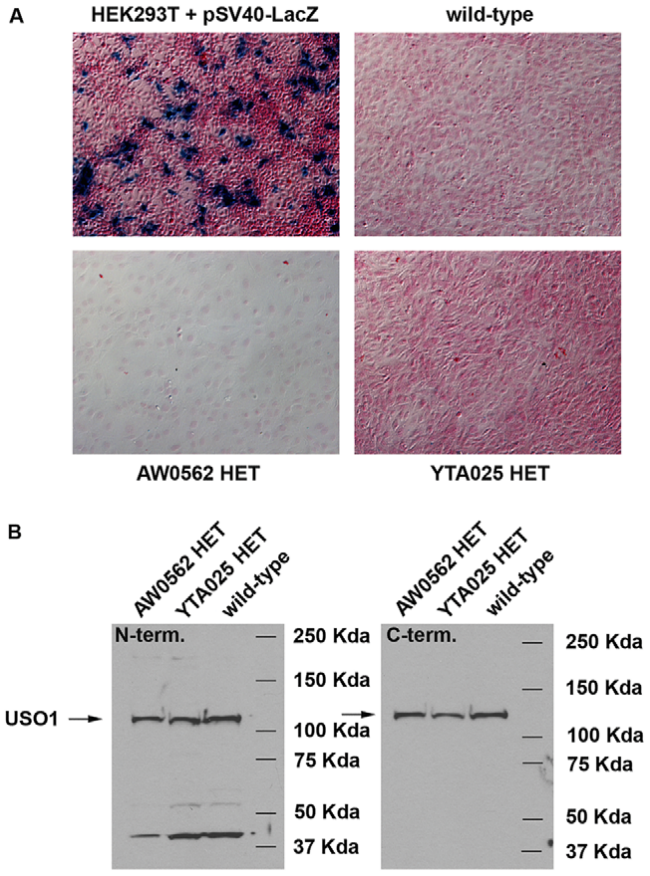
The *Uso1* gene trap allele does not produce a functional polypeptide. **A**) Photomicrographs of X-GAL stained HEK293T cells that had been transiently transfected with the Beta-galactosidase expression vector pSV40-LacZ (positive control) and X-GAL stained primary skin fibroblasts from wild-type, heterozygous (HET) AW0562 GT, and HET YTA025 GT mice. No X-GAL staining was observed in WT or heterozygous GT fibroblasts. **B**) Immunoblots of SDS-PAGE separated cell lysate extracted from wild-type, HET AW0562 GT and HET YTA025 GT fibroblasts. Left panel: an anti-USO1 antibody whose epitope is amino-terminal (N-term.) to the site of the USO1-Beta-Geo fusion detects full-length USO1 protein (arrow) in all lysates. No unique band representing a USO1-Beta-Geo fusion protein is observed in either heterozygous GT fibroblast lysate, although smaller bands of indeterminate origin are detected in wild-type and GT lysates. Right panel: an anti-USO1 antibody whose epitope is carboxyl-terminal (C-term.) to the site of the USO1-Beta-Geo fusion detects full length USO1 protein (arrow) in all lysates.

### Primary skin fibroblast cultures

Newborn pups from a heterozygous *Uso1* GT mating were euthanized and skinned. The skin was washed in PBS and diced into small pieces. Skin fragments were placed in 6-well plates and dried for 30 minutes to allow the skin to attach to the plastic. The adherent fragments were then cultured in 0.5 ml of DMEM/10% FBS. Primary skin fibroblast outgrowths were observed 5–7 days after plating. When the primary cultures reached 50% confluency, cells were trypsinized and transferred to a 25 cm^2^ flask for expansion.

**Figure 3:**
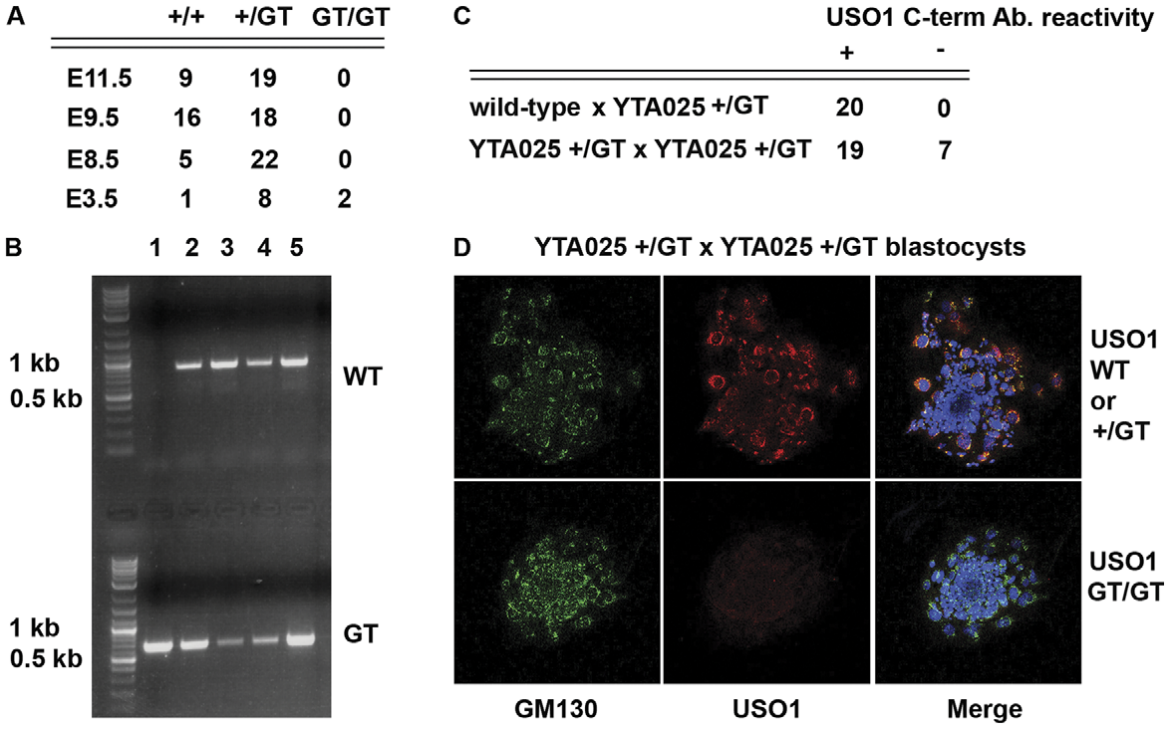
Fetal death occurs by E8.5 in embryos that are homozygous for the *Uso1* GT alleles. **A**) Table indicating the frequencies of genotypes in fetuses/blastocysts recovered from heterozygous *Uso1* GT mating pairs. Anticipated genotypes included WT (+/+), heterozygous GT (+/GT), and homozygous GT (GT/GT). No homozygous GT fetuses were observed at E11.5, E9.5, and E8.5. In contrast, 2 out of 11 E3.5 blastocysts were homozygous for the GT. **B**) Genotypes of E3.5 blastocysts obtained from a heterozygous AW0562 GT mating pair. One blastocyst was homozygous for the GT (lane 1), while 4 others were heterozygous. **C**) Table indicating the frequencies of immuno-detectable USO1 protein in cultured E3.5 blastocysts from wild-type x heterozygous YTA025 GT and heterozygous YTA025 GT x heterozygous YTA025 GT mating pairs. Immuno-detection was performed using an antibody that recognizes an epitope in the USO1 carboxyl-terminal domain. **D**) Photomicrograhs of double immunofluorescence images of cultured E3.5 blastocysts recovered from a heterozygous YTA025 GT mating pair. Antibodies that recognize epitopes in the USO1 carboxyl-terminal domain (red fluorescence) or the Golgi protein GM130 (green fluorescence) were employed. DAPI staining was used to mark cell nuclei (blue fluorescence). The upper panels depict fluorescence patterns that represent a blastocyst that is either wild-type (+/+) or heterozygous for the GT allele (+/GT). The lower panels depict fluorescence patterns that represent a blastocyst that is homozygous for the *Uso1* GT allele (GT/GT).

### Identification of Uso1-gene trap mRNA transcripts

Splicing of the GT into the *Uso1* mRNA was confirmed by RT-PCR using the sequence tag information provided by the International Gene Trap Consortium. Briefly, total RNA was extracted from primary skin fibroblasts cultures of heterozygous GT mice using Trizol following the manufacturer's recommendation (Invitrogen). Two µg of total RNA was reverse transcribed using a combination of oligo dT and random hexamers (Advantage RT-PCR kit, Clontech). Transcript containing the spliced GT allele was detected by PCR using a GT vector-specific reverse primer (5′-AGTATCGGCCTCAGGAAGATCG-3′) in combination with a forward primer in *Uso1* exon 10 (5′-TTGTGCGGGTACTGGTATCTCCCAC-3′) for AW0562 and in *Uso1* exon 12 (5′-GTGCCGTGCTCTCTGTTTCCGTG-3′) for YTA025. Wild-type allele transcript was detected by PCR using the aforementioned forward primers in combination with a reverse primer located in *Uso1* exon 13 (5′-CATAAGCCTTGGACCAACTGCTCTTC-3′). 36 cycles of PCR were performed using Platinum Taq polymerase (Invitrogen), an annealing temperature of 60°C, and an extension time of 2 minutes.

**Figure 4:**
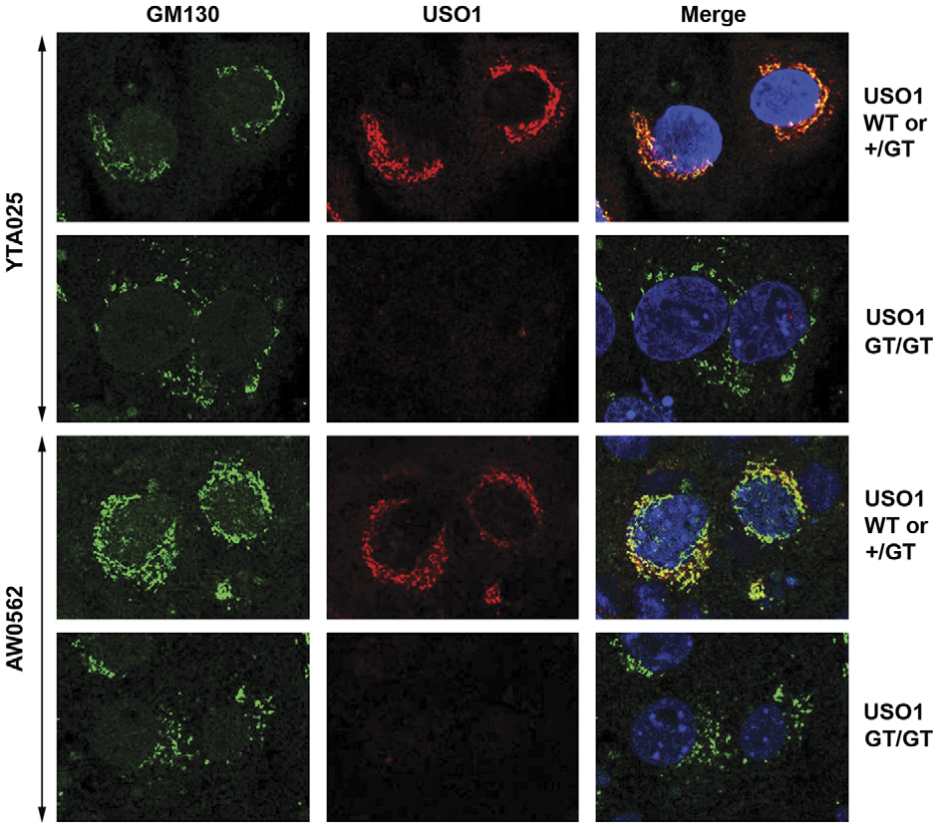
Blastocysts that are homozygous for a *Uso1* GT allele have a dispersed Golgi architecture. Confocal laser scanning double immunofluorescence images (magnification 400x) of cells within cultured E3.5 blastocysts that were recovered from heterozygous *Uso1* GT mating pairs. Antibodies recognizing epitopes in the USO1 carboxyl-terminal domain (red fluorescence) or the Golgi protein GM130 (green fluorescence) were used. DAPI staining was used to mark cell nuclei (blue fluorescence). In cells from blastocysts containing immuno-detectable USO1, GM130 localizes near the cell nuclei, overlapping with USO1 localization. In contrast, in cells from blastocysts that lack immuno-detectable USO1 protein, GM130 does not localize near the nucleus but is more dispersed throughout the cytoplasm.

### Genotyping mice for the Uso1 GT and wild-type alleles

Genotyping primers for the GT and wild-type alleles were designed after the specific insertion site of each GT was determined. Insertion sites were identified by performing long range PCR with a forward primer in the *Uso1* exon immediately upstream of the spliced GT exon, and a reverse primer (5′-GGAACAGGTATTCGCTGGTCACTTC-3′) contained within the GT vector. The forward primer for AW0562 line was in exon 10 (5′-TTGTGCGGGTACTGGTATCTCCCAC-3′ and the forward primer for the YTA025 line was in exon 12 (5′-GTGCCGTGCTCTACTGTTTCCAGTG-3′). Thirty-six cycles of PCR were performed using 500 ng of genomic DNA as template with Pfu Ultra polymerase (Applied Biosystems) at an annealing temperature of 60°C and an extension time of 7 minutes. Resulting amplimers were cloned using the TOPO-Zero-Blunt kit (Invitrogen) and Sanger sequenced.

Sequence information regarding the genomic DNA insertion site was then used to design new reverse primers, that when coupled with the original forward primer for each gene-trap line would generate PCR amplimers that were reliable for genotyping. The new reverse primer for the AW0562 GT allele was (5′-TACCAGACTCTCCCATCCACTACTC-3′) and for the YTA025 GT allele was (5′-CTAGAGTCCAGATCTGCGATAACTTC-3′). Reverse primers located downstream of each GT insertion site (5′-TCTGAAATAACTCAAGGTGGTTTGC-3′ for AW0562, and 5′-GTTACCTGTTGCTGCAAGCAGACAG-3′ for YTA025) were used to amplify the wild-type *Uso1* allele. A 60°C or 55°C annealing temperature was used when genotyping the AW0562 or YTA025 mice, respectively.

### X-gal staining to identify Beta-galactosidase activity

Primary skin fibroblasts and HEK293T (Human embryonic kidney cells, ATCC CRL1573) cells were plated onto 8-chamber culture slides (BD Biosciences). Upon reaching confluence, cells were washed with PBS and fixed in ice cold X-Gal fixative (PBS containing 0.2% glutaraldehyde, 5 mM EDTA and 2 mM MgCl_2_) for 10 minutes. Subsequently cells were washed 3x for 5 minutes with 0.5 ml wash solution (PBS containing 2 mM MgCl_2_ and 0.02% NP-40). X-gal staining was performed overnight in the dark (X-gal staining solution: PBS containing 5 mM Potassium-ferro-cyanide, 5 mM Potassium-ferri-cyanide, 2 mM MgCl_2_, 0.02% NP-40 and 2 mg/ml X-Gal). Cells were subsequently washed 3x with PBS and kept in PBS at 4°C. As a positive control for Beta-galactosidase activity the HEK293T cells were transfected with 0.5 µg of pSV40-LacZ (Promega).

### Immuno-detection of USO1 in cell lysate

Primary skin fibroblasts were lysed in RIPA buffer (Sigma) containing 1x EDTA free protease inhibitor cocktail (Thermoscientific) for 10 minutes on ice. One ml of lysis buffer was used to lyse fibroblasts collected from a confluent 75 cm^2^ culture flask. Lysates were then cleared of debris by centrifugation (16,100×g, 2 min). The protein concentration in each lysate was measured using the Bradford assay (Quick Start Bradford Dye reagent, Biorad) and RIPA buffer was then added to equalize the protein concentration across all lysates. Equal amounts of lysates were subsequently separated on a NUPAGE 3–8% Tris-Acetate gel (Invitrogen) and transferred overnight at 15 V onto a PVDF membrane (Invitrogen). Immunodetection of USO1 was performed using the Western breeze system (Invitrogen). An amino terminal anti-USO1 antibody (NB100-74483; Novus Biologicals) and a carboxyl-terminal USO1 antibody (13509-1-AP; Proteintech) were each used at a 1/1000 dilution.

### Retrieval of blastocysts from GT breeding pairs

Heterozygous GT breeding pairs were checked daily for mating by identification of vaginal plugs. When a vaginal plug was observed, the female was euthanized 72 hrs later, the uterus was removed and placed in a 60 mm dish containing 1 ml of M2 medium (Sigma), and the uterine horns were flushed using a 20 gauge needle with 0.5 ml of pre-warmed (37°C) M2 medium to obtain blastocysts. Blastocysts were identified microscopically, retrieved with a 0.8–1.10×100 mm capillary tube (Kimax), and placed individually into different gelatin-coated chambers filled with 0.2 ml of blastocyst medium (DMEM/15% FBS/non-essential amino acids; Invitrogen). Eight-chamber culture slides (BD Biosciences), pre-coated with 0.1% gelatin (Sigma) for 30 minutes at room temperature, were used. DNA was extracted from individual blastocysts after 3 days of culture (Arcturus PicoPure DNA extraction kit, Applied Biosystems) and used for WT and GT allele genotyping.

### Immuno-detection of USO1 and GM-130 in cultured blastocysts

After 3 days in culture, blastocysts were washed with 0.5 ml PBS and fixed to the glass slide with 0.5 ml of 4% paraformaldehyde for 20 minutes at room temperature. Cells were subsequently washed twice with PBS, twice with 0.1M NH_4_Cl and twice with PBS. Primary antibody incubation was performed overnight at 4°C in PBS containing 5% FBS, 2% BSA and 0.1% Saponin. Cells were washed 3x with 0.5 ml PBS and incubated with secondary antibody in PBS for 30 minutes at room temperature. Cells were subsequently washed 3x with 0.5 ml PBS and mounted in DAPI Fluoromount G (Southern Biotech). Primary antibodies were used in a 1/1,000 dilution and secondary antibodies were used in a 1/10,000 dilution. Primary antibodies used were mouse anti-GM130 (610822, BD Transduction laboratories) and rabbit anti-USO1 (13509-1-AP, Proteintech). Secondary antibodies used were Cy3 anti-rabbit IgG (XG-6157cy3, ProScience) and Fluorescein anti-mouse IgG (XR-9760, ProScience). Fluorescence images were obtained using a NikonRi1 camera mounted to a Nikon Eclipse 80i microscope. Confocal laser scanning microscopy was performed using the Zeiss LSM 780 system. Mutant and control pictures were equally adjusted for brightness and contrast using Adobe Photoshop CS3.

## Results

### Mice heterozygous for the AW0562 or YTA025 GT allele are viable and fertile

Mice heterozygous for a GT within *Uso1* (Figure 1A, B) were generated using the AW0562 and YTA025 ES cell lines. Sequence tag information obtained by RT-PCR using ES cell RNA had previously suggested that the AW0562 GT spliced to *Uso1* exon 10 and the YTA025 GT spliced to *Uso1* exon 12 (www.genetrap.org). We confirmed these results in heterozygous GT mice using RNA extracted from primary skin fibroblast cultures (Figure 1C). This led us to hypothesize the GT had inserted into intron 11 in the AW0562 cell line and into intron 13 in the YTA025 cell line. We confirmed our hypotheses by using forward primers located in exons 10 or 12 of *Uso1* and a reverse primer located in the GT to PCR amplify genomic DNA from mice derived from AW0562 and YTA025 ES cells, respectively. We observed a PCR amplimer ∼5000 bp in length for AW0562 and an amplimer ∼3,000 bp in size for YTA025 (data not shown). Sequence analyses of these amplimers indicated that the AW0562 GT had inserted 541 bp downstream of exon 10 and the YTA025 GT had inserted 340 bp downstream of exon 12. Based on this information, we designed new primers to facilitate genotyping the wild-type and GT alleles (Figure 1D). Male and female heterozygous GT mice were phenotypically indistinguishable from wild-type mice and were able to transmit the GT allele to 50% of their offspring.

### Each Uso1 GT creates a functional null allele

The GT vectors used to generate AW0562 and YTA025 were designed to generate a USO1-Beta-Geo fusion protein. The mRNA transcript encoding this fusion protein was detected by RT-PCR (Figure 1C). Therefore, we looked for expression of chimeric protein by performing X-gal staining on primary skin fibroblasts derived from wild-type, heterozygous AW0562 GT, and heterozygous YTA025 GT mice. We saw no evidence of Beta-Geo activity (Figure 2A). To exclude the possibility that the GT alleles produced fusion proteins that were enzymatically inactive, we performed SDS-PAGE and western blotting on cell lysates from wild-type and heterozygous GT fibroblasts. To detect endogenous and chimeric USO1 protein, we used anti-USO1 antibody against an epitope that is amino-terminal of the USO1-Beta-Geo fusion. The chimeric protein should differ in size from wild-type protein. However, we only detected a protein band corresponding in size to wild-type USO1 protein in the heterozygous GT lysates (Figure 2B). This same sized wild-type band was detected using an anti-USO1 antibody generated against an epitope that is carboxyl-terminal of the USO1-Beta-Geo fusion (Figure 2B). These data suggest that each GT allele fails to produce a stable mRNA or protein product, and is therefore a functional null allele.

### Embryos homozygous for Uso1 GT alleles die before E8.5

To determine the *in vivo* phenotype of *Uso1* inactivation, we crossed heterozygous GT mice and looked at their offspring for GT homozygotes. No offspring were homozygous for either the AW0562 or YTA025 GT allele, suggesting that inactivation of *Uso1* causes embryonic lethality. To more precisely determine the stage at which homozygous GT embryos die, we recovered fetuses from heterozygous matings at E11.5, E9.5 and E8.5. At each time point no homozygous GT fetuses were recovered (Figure 3A). However, we observed several deciduas containing no identifiable embryos at E8.5 and E9.5, indicating that some embryos had died after implantation.

### Retrieval of blastocysts homozygous for the Uso1 GT alleles

To determine whether inactivation of *Uso1* is compatible with fertilization and early cleavage events, blastocysts were recovered from GT heterozygous matings. The blastocysts were placed in individual chambers of 8-well culture slides and kept in culture. Between 48 and 72 hours, the blastocysts attached to the glass slide and giant trophoblasts cells and smaller inner mass cells could be observed (Figure 3D). After 3 days, DNA was extracted from the attached blastocysts and used for genotyping. Blastocysts homozygous for both AW0562 and YTA025 were retrieved (Figure 3A and 3B and data not shown). This indicates that inactivation of *Uso1* does not affect fertilization or the first stages of embryonic development.

We needed DNA from the entire blastocyst to determine its genotype by PCR. Therefore, to independently demonstrate that blastocysts lacking USO1 could be recovered at E3.5, we immunofluorescently double-labeled blastocysts using an antibody against an epitope in USO1 that is carboxyl-terminal to the USO1-Beta-Geo fusion and an antibody against another Golgi protein GM130. All 20 blastocysts retrieved from matings between wild-type and *Uso1* GT heterozygous mice had immuno-detectable USO1 and GM130 protein (Figure 3C and data not shown). In contrast, 7 of 19 blastocysts retrieved from matings between heterozygous *Uso1* GT mice lacked immuno-detectable USO1 but had immuno-detectable GM130 (Figure 3D).

The data presented in figure 3A and 3C also show that *Uso1* GT homozygous blastocysts are present in a Mendelian ratio. Nine *Uso1* GT homozygous blastocysts out of a total of 37 blastocysts were recovered from *Uso1* GT heterozygous matings, indicating that *Uso1* GT homozygous blastocysts do not die before E3.5.

### Disrupted Golgi apparatus in blastocyst cells homozygous for the Uso1 GT

*In vitro* knock down of *Uso1* expression using siRNA indicated that USO1 is needed to maintain the Golgi structure [12]. To test whether USO1 maintains the Golgi structure *in vivo*, we performed confocal laser scanning microscopy to examine the distribution of GM130 protein in blastocysts that contained or lacked immuno-detectable USO1. Blastocysts obtained from matings of heterozygous AW0562 GT or heterozygous YTA025 GT mice gave the same results. Blastocysts that lacked immuno-detectable USO1 exhibited dispersal of GM130 containing foci throughout the cytoplasm of all trophoblast cells, consistent with a disruption in Golgi apparatus structure when USO1 is absent (Figure 4).

## Discussion

An important step in the fusion of a membrane trafficking intermediate with its acceptor compartment is its recognition and capture by tethering factors. The golgin protein family represents a subset of these tethering factors. In addition to vesicle capturing, siRNA studies in cultured cells have shown that golgins also play a central role in the maintenance of the Golgi structure [8]. A number of different golgins can be found located at the Golgi apparatus. In each case of the golgins TMF, USO1, GM130, GRASP-55, GRASP-65 and GMAP-210, knock down of their expression *in vitro* resulted in a fragmentation of the Golgi apparatus and the dispersal of Golgi marker proteins throughout the cytoplasm [9], [12], [16], [17], [18].

Given the importance of the Golgi apparatus to the efficient modification and trafficking of cargoes, mice lacking proteins that maintain Golgi structure would have been predicted to exhibit early embryonic lethality. However, mice lacking GMAP-210 do not display early embryonic lethality. With the exception of a significant disruption of skeletal growth, most other organs appear to develop normally in mice lacking GMAP-210 [7], [19]. Recently, it was reported that *in vivo* inactivation of another ubiquitously expressed golgin GIANTIN also resulted in a tissue specific phenotype, mainly impairing skeletal development [10]. Functional compensation or functional overlap among golgins may occur across many cell types, thereby explaining why deficiency of individual golgins can cause narrower *in vivo* phenotypes than predicted from *in vitro* knockdown experiments [8], [20].

In contrast to the tissue-specific *in vivo* phenotypes observed when GMAP-210 and GIANTIN are deficient, *in vivo* deficiency of USO1 causes early embryonic lethality. *Uso1* null mice died between E3.5 and E8.5. Cultured *Uso1* deficient blastocysts were able to attach to the glass slides and exhibit outgrowth of giant trophoblasts and inner mass cells. However, the structure of the Golgi apparatus within trophoblast cells was fragmented, similar to what has been observed when USO1 is depleted *in vitro* [12].

Our present study shows that USO1 is an essential membrane trafficking protein. Given the central role of membrane trafficking in cellular function, one might have expected that *Uso1* GT homozygous embryos would not have been able to even reach the blastocyst stage, let alone be viable for at least three more days in culture. Several studies have shown that the severity of the effect of siRNA knock down of USO1 on intracellular protein transport depends on the protein examined, with intracellular transport of certain proteins only minimally affected [21], [22]. It is thus possible that in the absence of USO1, enough essential proteins are still being correctly transported for the fertilized oocyte to progress towards the blastocyst stage but not for further embryonic development after implantation. An alternative explanation is, that retention of maternally derived wild type *Uso1* transcript or USO1 protein masks the lethality of *Uso1* inactivation. *Uso1* transcript is present in oocytes and early cleavage stage embryos [23] and several studies have demonstrated that maternal transcripts can persist up to the blastocyst stage [23], [24], [25].

In conclusion, we have demonstrated that USO1 is indispensable during early embryonic mammalian development. As a consequence, studying the role of USO1 in individual tissues will require a conditional knock-out approach.
